# Valproic Acid Enhanced Temozolomide-Induced Anticancer Activity in Human Glioma Through the p53–PUMA Apoptosis Pathway

**DOI:** 10.3389/fonc.2021.722754

**Published:** 2021-10-01

**Authors:** Hong-Chieh Tsai, Kuo-Chen Wei, Pin-Yuan Chen, Chiung-Yin Huang, Ko-Ting Chen, Ya-Jui Lin, Hsiao-Wei Cheng, Yi-Rou Chen, Hsiang-Tsui Wang

**Affiliations:** ^1^ Department of Neurosurgery, Linkou Chang Gung Memorial Hospital, Taoyuan, Taiwan; ^2^ School of Traditional Chinese Medicine, Chang Gung University, Taoyuan, Taiwan; ^3^ Department of Neurosurgery, New Taipei Municipal TuCheng Hospital, Chang Gung Memorial Hospital, New Taipei City, Taiwan; ^4^ Neuroscience Research Center, Linkou Chang Gung Memorial Hospital, Taoyuan, Taiwan; ^5^ School of Medicine, Chang Gung University, Taoyuan, Taiwan; ^6^ Department of Neurosurgery, Keelung Chang Gung Memorial Hospital, Keelung, Taiwan; ^7^ Institute of Pharmacology, College of Medicine, National Yang-Ming University, Taipei, Taiwan; ^8^ Institute of Pharmacology, College of Medicine, National Yang Ming Chiao Tung University, Taipei, Taiwan; ^9^ Institute of Food Safety and Health Risk Assessment, National Yang Ming Chiao Tung University, Taipei, Taiwan; ^10^ Doctor Degree Program in Toxicology, Kaohsiung Medical University, Kaohsiung, Taiwan

**Keywords:** glioblastoma, temozolomide, valproic acid, p53, PUMA, apoptosis

## Abstract

Glioblastoma (GBM), the most lethal type of brain tumor in adults, has considerable cellular heterogeneity. The standard adjuvant chemotherapeutic agent for GBM, temozolomide (TMZ), has a modest response rate due to the development of drug resistance. Multiple studies have shown that valproic acid (VPA) can enhance GBM tumor control and prolong survival when given in conjunction with TMZ. However, the beneficial effect is variable. In this study, we analyzed the impact of VPA on GBM patient survival and its possible correlation with TMZ treatment and *p53* gene mutation. In addition, the molecular mechanisms of TMZ in combination with VPA were examined using both p53 wild-type and p53 mutant human GBM cell lines. Our analysis of clinical data indicates that the survival benefit of a combined TMZ and VPA treatment in GBM patients is dependent on their *p53* gene status. In cellular experiments, our results show that VPA enhanced the antineoplastic effect of TMZ by enhancing p53 activation and promoting the expression of its downstream pro-apoptotic protein, PUMA. Our study indicates that GBM patients with wild-type *p53* may benefit from a combined TMZ+VPA treatment.

## Introduction

Glioblastoma (GBM), the most common and most lethal type of brain tumor, accounts for 50% of malignancies in the intrinsic central nervous system and has the highest loss of potential life years compared to other cancers (1–3). Standard treatment includes surgical excision and concomitant chemoradiotherapy with temozolomide (TMZ), followed by TMZ chemotherapy. Recent studies in molecular biology have shown that, despite similarities in histological appearances, GBM harbors significant genetic, epigenetic, and gene expression heterogeneities both interpersonally and intratumorally (4–6). The heterogeneous genetic background of GBM patients results in variable sensitivities of cancer cells to TMZ treatment and, thus, differential clinical outcomes. Hypermethylation at the promoter of *O* ^6^-methylguanine DNA methyltransferase (*MGMT*), a DNA repair gene, is associated with increased sensitivity to TMZ treatment and improved patient survival (7–11). Isocitrate dehydrogenase 1 (*IDH1*) mutant GBM patients have more favorable outcomes, partly due to the enhanced sensitivity to TMZ chemotherapy (12, 13). On the other hand, a dysfunctional p53 DNA response pathway is associated with TMZ resistance (10, 14, 15). Previous studies have shown that somatic alterations that deregulate p53 were found in 85%–90% of GBM tumors, including 27.9% of *p53* gene mutations or deletions (16). Furthermore, *p53* mutation often co-occurs with *IDH1* and *ATRX* mutations, which are critical markers defining GBM molecular classification (17, 18). These molecular alterations have significant clinical implications in that they not only define radically different subgroups of GBM but also significantly affect tumor susceptibility to treatment and, thus, patient prognosis (19–21).

Valproic acid (VPA) is an anti-epileptic drug that is widely used to treat or prevent perioperative seizures associated with GBM (22). In addition to its anti-epileptic activity, it has been shown in some retrospective studies that VPA was capable of improving GBM patient survival when given in conjunction with TMZ (23–26). However, the results of these studies are inconsistent. Multiple mechanisms of the anticancer action of VPA have been proposed, including inhibition of histone deacetylase (HDAC), alteration in the chromatin structure, disruption of DNA repair pathways or redox regulation, and induction of autophagy (27–30). Several reports have shown that VPA induces p53-dependent radiosensitization and chemosensitization *in vitro* and *in vivo* (31–34). In the present study, we attempted to identify GBM genetic alterations that are associated with favorable outcomes of VPA treatment in GBM patients and decipher the underlying mechanism. In clinical samples, GBM patient survival with respect to VPA treatment and *p53* gene status was investigated. Furthermore, the effect of TMZ alone or in combination with VPA on GBM cancer cells was examined using both p53 wild-type and p53 mutant human GBM cell lines, and its underlying molecular mechanisms were examined.

## Materials and Methods

### Patient Data Collection

The institutional database of Linkou Chang-Gung Memorial Hospital was used to identify patients who underwent surgical excision of GBM between January 2015 and December 2017 following guidelines approved by the IRB board (IRB# 201701979B0). Patients who underwent standard treatment for newly diagnosed GBM, which included surgical excision, TMZ combined chemoradiotherapy (CCRT), and oral TMZ chemotherapy, and patients who underwent surgical excision for recurrent GBM were included. Patients who underwent biopsy only, did not undergo surgical excision, had a diagnosis based on imaging only, did not have histopathologically proven GBM, and did not undergo standard treatment for newly diagnosed GBM were excluded. A total of 166 patients were recruited. The medical records of these patients were reviewed and followed up until December 31, 2019. Clinical details, including immunohistochemical (IHC) staining of specific markers such as MGMT, the patient’s seizure status, date of birth, date of diagnosis, date of operation, and the recorded date of disease progression or death, were recorded. Residual tumor samples were obtained from the tumor bank.

### *p53* and *IDH1* Mutation Analysis

Genomic DNA was extracted from frozen tissues, exon 4 of *IDH1* and *IDH2* or exons 4–9 of *p53* were PCR amplified from tumor DNA, and mutations were analyzed by sequencing analysis. The primers used in PCR and sequencing are shown in **Table 1**. All mutations were confirmed by sequencing both DNA strands.

**Table 1:** The sequencing primers of exon 4 of IDH1 and IDH2 or exon 4-9 of p53.

*IDH1* exon 4	Forward: TGAGCTCTATATGCCATCACTGCA
	Reverse: CAATTTCATACCTTGCTTAATGGG
*IDH2* exon 4	Forward: GTCTGGCTGTGTTGTTGCTTG
	Reverse: CAGAGACAAGAGGATGGCTAGG
*p53* exon 4	Forward: TGAGGACCTGGTCCTCTGAC
	Reverse: AGAGGAATCCCAAAGTTCCA
*p53* exons 5–6	Forward: TGTTCACTTGTGCCCTGACT
	Reverse: TTAACCCCTCCTCCCAGAGA
*p53* exon 7	Forward: AGGCACACTGGCCTCATCTT
	Reverse: TGTGCAGGGTGGCAAGTGGC
*p53* exons 8–9	Forward: TTGGGAGTAGATGGAGCCT
	Reverse: AGTGTTAGACTGGAAACTTT

Sequencing of exon 4 of IDH1 and IDH2 was based on previously described (35) and sequencing of exon 4-9 of the TP53 gene was carried out following the method from IARC TP53 database (R20).

### Cell Culture and Treatment

The GBM cell lines U87, DBTRG-05MG, U118MG, and LN229 were purchased from ATCC (Manassas, VA, USA). U87 cells were grown in minimum essential medium (MEM) supplemented with 10% fetal bovine serum (FBS) at 37°C in an atmosphere of 5% CO_2_. DBTRG-05MG cells were grown in RPMI-1640 supplemented with 10% FBS at 37°C in an atmosphere of 5% CO_2_. U118MG and LN229 cells were maintained in Dulbecco’s modified Eagle’s medium (DMEM) supplemented with 10% FBS at 37°C in an atmosphere of 5% CO_2_. The *p53* status of these cell lines was confirmed using DNA sequencing analysis (**Supplementary Figure S7**). Cells at 70% confluency were washed with phosphate-buffered saline (PBS) and treated with TMZ (0–10 mM) and/or VPA (2.5 mM) in complete culture medium at 37°C in the dark.

### RNA Interference

Knockdown of *p53*, *E2F1*, or *HDAC2* in GBM cells by RNA interference with human p53 siRNA (Sigma, St. Louis, MO, USA), E2F1 Silencer Select siRNA (/N4390824; Life Technology Corporation, Carlsbad, CA, USA), or HDAC2 Silencer Select siRNA (AM51331; Life Technology Corporation) was carried out according to the manufacturer’s protocol using GenMute siRNA Transfection Reagent (SignaGen Laboratories, Frederick, MD, USA). The sequence of the p53 siRNA targeting p53 mRNA (NM_000546.5) was GACUCCAGUGGUAAUCUAC, and siRNA was synthesized by Sigma (St. Louis, MO, USA). p53 siRNA, E2F1 siRNA, HDAC2 siRNA, or control siRNA was transfected at a final concentration of 30 nM for 24 h, followed by TMZ or VPA treatment as described above. Western blotting and reverse transcription PCR (RT-PCR) analyses were used to verify the efficiency of transfection.

### Cell Viability Assay

GBM cells were plated in 96-well culture plates and incubated in a humidified chamber at 37°C and 5% CO_2_ overnight before drug treatment. Cell viability was assessed 24 h after the addition of the drugs. The culture medium was then removed from each well and replaced with 150 μl of MTT (3-[4,5-dimethylthiazol-2-yl]-2,5 diphenyl tetrazolium bromide; 0.5 mg/ml) in complete medium. The cells were incubated for 2 h at 37°C. Formazan crystals formed in cells were dissolved in 50 μl of dimethyl sulfoxide (DMSO). The absorbance of the samples at 570 nm was measured using a microplate reader. The results were evaluated by measuring the optical density of the MTT solution at 570 nm.

### Western Blot Analysis

The cell lysate was prepared by sonication in RIPA buffer with protease and phosphatase inhibitor cocktails. Proteins (30 µg) were separated by 8%–15% sodium dodecyl sulfate–polyacrylamide gel electrophoresis and transferred to polyvinylidene difluoride membranes, blocked in 5% milk, and incubated overnight at 4°C in primary antibodies including PARP (1:1,000; #9542, Cell Signaling, Danvers, MA, USA), E2F1 (1:1,000; #3742, Cell Signaling, Danvers, MA, USA), p53 (DO-1) (1:1,000;, cat. no. OP43, Calbiochem, San Diego, CA, USA), PUMA (1:1,000; #12450, Cell Signaling, Danvers, MA, USA), caspase 3 (1:1,000; #9662, Cell Signaling, Danvers, MA, USA), and caspase 9 (1:1,000; #9502, Cell Signaling, Danvers, MA, USA), followed by the respective anti-IgG secondary antibodies (1:3,000; Millipore, Burlington, MA, USA) for 1 h at room temperature. Membranes were developed for visualization and photography using enhanced chemiluminescence (ECL) (Millipore Corporation, Billerica, MA, USA). Optical band densities were quantified using ImageJ software, and the results were analyzed using Excel software.

### Flow Cytometry Analysis of the Cell Cycle Phases

After harvesting, the cells were washed twice in ice-cold PBS and fixed in ice-cold 70% ethanol for 30 min or overnight at 4°C. The cells were washed in PBS and digested with DNase-free RNase A (50 U/ml) at 37°C for 30 min. Before flow cytometry analysis, the cells were resuspended in 500 μl propidium iodide (PI, 10 μg/ml; Sigma, St. Louis, MO, USA) for DNA staining. PI staining was used to measure the cell cycle status using a Becton-Dickinson FACScan instrument (Franklin Lakes, NJ, USA) and the Cell Quest software.

### Quantitative Real-Time RT-PCR

Total RNA was isolated from the harvested cells using the TRIzol*^®^* Reagent (Thermo Fisher Scientific, Waltham, MA, USA) and underwent reverse transcription using RevertAid Reverse Transcriptase (Thermo Fisher Scientific) according to the manufacturer’s instructions. Subsequent real-time RT-PCR analysis of cDNA was performed in triplicate using SYBR green dye on the StepOnePlus™ Real-Time PCR System (Applied Biosystems, Waltham, MA, USA). The primer sequences are shown as follows (5'–3'): PUMA, CCTGGAGGGTCCTGTACAATCTC; GCAGGCACCTAATTGGGCTC; GAPDH: CCGTCTAGAAAAACCTGCC; GCCAAATTCGTTGTCATACC. To calculate the relative mRNA expression, GAPDH was used as an internal control for all quantitative RT-PCRs and compared with the control groups.

### Statistical Analyses

Student’s *t*-tests were used to determine statistical significance, and two-tailed *p*-values are shown. A minimum of three independent replicate experiments were performed to justify the use of statistical tests. Survival and progression-free survival were analyzed using Kaplan–Meier survival analysis, and the log-rank test was used for comparisons between two groups. Multivariate analysis was performed using Cox regression analysis. *P* < 0.05 was considered statistically significant. All statistical analyses were performed using SPSS software version 20.0.

## Results

### TMZ Combined with VPA Is Associated With Improved Survival in GBM Patients With Wild-Type *p53*

To evaluate the effect of VPA on the survival of GBM patients, we retrospectively reviewed patients diagnosed with primary GBM from 2015 to 2017 who underwent curative excisional surgery. Some of these patients underwent more than one surgical excision during these 2 years. For the purpose of analysis, we only included each patient’s first operation. The demographic data are shown in **Table 2**. Of all 166 patients, 139 underwent surgery for newly diagnosed GBM and 27 underwent surgery for recurrent tumors. All newly diagnosed GBM patients underwent standard treatment, including surgical excision, combined chemoradiotherapy with TMZ, and TMZ chemotherapy. All recurrent GBM patients received avastin treatment after surgical excision. The median survival of all patients combined was 15.20 ± 1.17 months, with 15.67 ± 1.42 months for newly diagnosed patients and 13.10 ± 2.34 months for recurrent GBM patients.

**Table 2:** Patient demographics and clinical characteristics of glioblastoma (GBM) patients.

		VPA treatment				*p*-value
		Short-term (no or <30 days)		Long-term (≥30 days)		
		Count	%	Count	%	
Age (years)	Mean ± SD	58.18 ± 14.61		54.98 ± 14.26		0.854
	<65	76	69.0	41	73.2	0.582
	≥65	34	31.0	15	26.8
Gender	Male	71	64.5	25	44.6	0.014*
	Female	39	35.5	31	55.4
Seizure status	Yes	41	37.2	35	62.5	0.002*
	No	69	62.8	21	37.5
New/recurrent	Newly diagnosed	88	80.0	51	91	0.068
	Recurrent	22	20.0	5	9

VPA, valproic acid.

*p < 0.05 (the chi-square statistic is significant at the 0.05 level).

**Figure 1:**
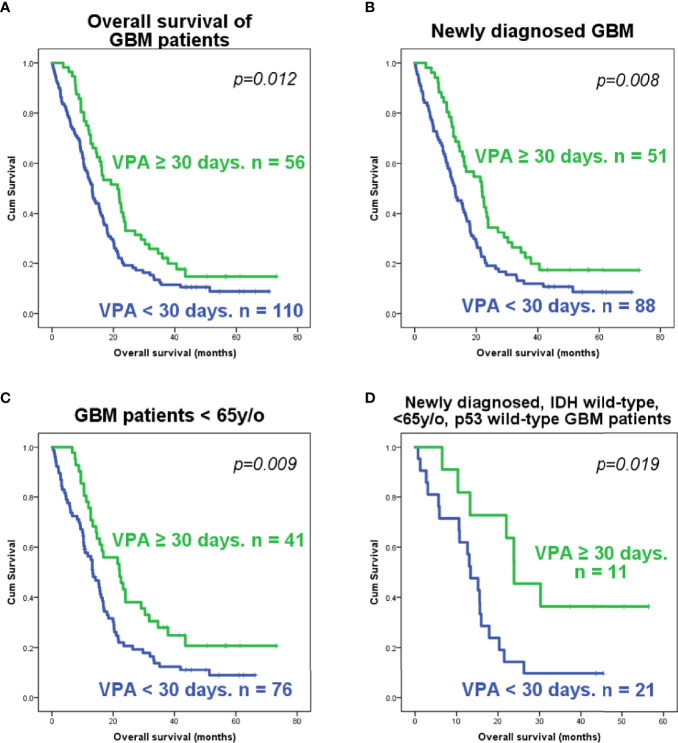
Kaplan–Meier analysis of the survival of glioblastoma (GBM) patients according to the valproic acid (VPA) treatment group. Survival plots of all GBM patients **(A)**, newly diagnosed GBM patients **(B)**, younger GBM patients (<65 years old) **(C)**, and younger (<65 years old), newly diagnosed GBM patients with wild-type *IDH1* and wild-type *p53* **(D)**. The *p*-value was calculated using the log-rank test in SPSS software.

Of these 166 patients, 51 out of 139 newly diagnosed GBM patients and 5 out of 27 recurrent GBM patients had VPA treatment for over 30 days. These patients were classified into the long-term VPA group. Patients who received long-term VPA treatment had significantly longer survival (**Figure 1A**), which is concordant with our previous report (36). Kaplan–Meier survival analyses showed that only long-term VPA treatment was associated with longer overall survival in the combined newly diagnosed and recurrent patients, while younger age (<65 years), seizure history, and long-term VPA treatment were associated with better survival in newly diagnosed GBM patients (**Figure 1B** and **Supplementary Figures S1A–C**). In the Cox regression analysis, none of these factors were independent prognostic factors (**Supplementary Tables S1** and **S2**). We further analyzed the characteristics of patient who underwent VPA treatment and their impact on the effects of VPA treatment. There were significantly more seizure patients and more female patients in the long-term VPA group (**Table 2**). The effect of VPA on median survival in the different groups is shown in **Supplementary Table S3**. Favorable outcomes with VPA treatment were observed in patients with newly diagnosed GBM (**Figure 1B**) and in patients who were under 65 years old (**Figure 1C**), but not in those with recurrent GBM (**Supplementary Figure S1D**) or older patients (≥65 years old) (**Supplementary Figure S1E**). The effect was not affected by the patient’s gender or seizure status.

To further elucidate the effects of VPA on GBM subtypes, we examined the IHC staining reports of these patients to identify the expression status of MGMT and study its correlation with the VPA treatment effect. Additionally, we tried to obtain surgical specimens from these patients and evaluated their *IDH1* mutation status. *MGMT* promoter methylation inhibits DNA repair gene expression and is significantly associated with improved patient survival and TMZ sensitivity. Of the 166 patients recruited, IHC staining was available in 92 patients. No correlation was found between the expression of MGMT determined by IHC and patient survival (**Supplementary Figure S2**). Additionally, VPA treatment did not result in a statistically significant overall survival benefit in either MGMT staining-positive or MGMT staining-negative patients (**Supplementary Figure S3**), although there appears to be a trend toward better survival in long-term VPA-treated MGMT staining-negative newly diagnosed GBM patients (**Supplementary Figure S3E**).

Since several previous studies have indicated that VPA affects tumor growth *via* p53-dependent pathways (31–34), we also checked the *p53* mutation status in these tissue samples. We were able to obtain 85 tumor tissue samples from these GBM patients. The demographic data and gene analysis results are shown in **Table 3**. The majority of these samples were *IDH1* wild type, *IDH2* wild type, and *p53* wild type. Ten samples out of these 85 had *IDH1* ^R132^ mutations, while none had *IDH2* mutations. Of the *p53* mutations identified in 11 tumors, 10 were missense mutations and one was a nonsense mutation, and all of them were identified as p53-inactivating mutations in the IARC TP53 database (R20) (37) (**Supplementary Table S4** and **Supplementary Figure S4**). The mutational status of *p53* was not significantly associated with the overall survival or progression-free survival of either newly diagnosed or recurrent GBM patients (**Supplementary Figure S5**). Further analysis revealed that VPA treatment is associated with improved survival in GBM patients who were under 65 years old and had newly diagnosed *IDH* wild-type and *p53* wild-type GBM (**Figure 1D**), but not in patients who were under 65 years old and had newly diagnosed *IDH* wild-type and *p53* mutant GBM, patients who were older than 65 years with newly diagnosed *IDH* wild-type GBM, and patients with newly diagnosed *IDH* mutant GBM, with recurrent GBM regardless of the *p53* or *IDH* mutational status, and with *p53* mutant or *IDH* mutant GBM regardless of age or recurrence status (**Supplementary Figure S6**).

**Table 3:** Patient demographics and clinical characteristics of 85 glioblastoma (GBM) patients whose tumor samples underwent genetic evaluation.

		VPA treatment				*p*-value
		Short-term (no or <30 days)		Long-term (≥30 days)		
		Count	%	Count	%	
Age (years)	Mean ± SD					
	<65	42	68.9	21	87.5	0.101^a^
	≥65	19	31.1	3	12.5
Gender	Male	38	62.3	12	44.6	0.335
	Female	23	37.7	12	50.0
Seizure status	Yes	21	34.4	18	75.0	0.001*
	No	40	65.6	6	25.0
New/recurrent	Newly diagnosed	45	73.8	20	83.3	0.409^a^
	Recurrent	16	26.2	4	16.7
Mutation status	*IDH1* mutant	6	9.8	4	16.7	0.458
	*IDH2* mutant	0	0	0	0	0.496
	*p53* mutant	7	11.5	4	16.7	0.496

VPA, valproic acid.

*p < 0.05 (the chi-square statistic is significant at the 0.05 level).

a Fisher’s exact test.

In conclusion, our results indicate that the survival benefit of VPA in GBM patients may be dependent on the patient’s age, *IDH* mutation status, and *p53* mutation status. Long-term VPA treatment may confer some degree of survival benefit in newly diagnosed and *p53* wild-type GBM patients who are under 65 years old.

### VPA Treatment Enhanced TMZ-Induced Cytotoxicity in GBM Cancer Cells in a p53-Dependent Manner

**Figure 2:**
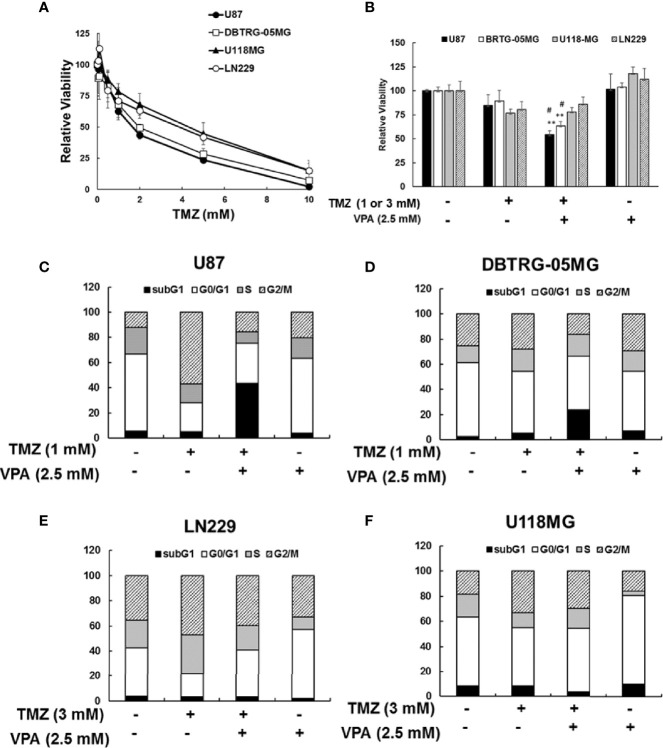
Cytotoxicity of temozolomide (TMZ) without or with valproic acid (VPA) in glioblastoma (GBM) cell lines. **(A)** Cytotoxicity of different dosages of TMZ (0–10 mM) for 24 h in p53 wild-type GBM cells (U87 and DBTRG-05MG) and p53 mutant GBM cells (U118MG and LN229). **(B)** Cytotoxicity of TMZ (1 or 3 mM) combined with VPA (2.5 mM) for 24 h in p53 wild-type GBM cells (U87 and DBTRG-05MG) and p53 mutant GBM cells (U118MG and LN229). Cytotoxicity was analyzed using the MTT assay, as described in *Materials and Methods*. Data are the mean ± SD. **p < 0.01 compared with cells without TMZ or VPA treatment; ^#^ *p* < 0.05 compared with cells with TMZ treatment. **(C, D)** Cell cycle analysis of TMZ (1 mM) combined with VPA (2.5 mM) for 24 h in p53 wild-type GBM cells (U87 and DBTRG-05MG). **(E, F)** Cell cycle analysis of TMZ (3 mM) combined with VPA (2.5 mM) for 24 h in p53 mutant GBM cells (U118MG and LN229). Cell cycle was analyzed using propidium iodide (PI) staining with flow cytometry, as described in *Materials and Methods*.

**Figure 3:**
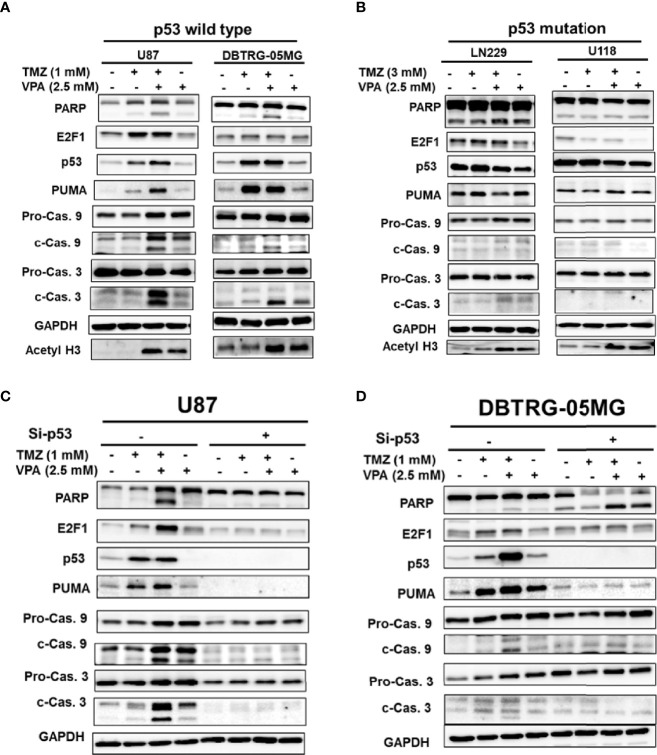
Temozolomide (TMZ) combined with valproic acid (VPA) enhanced cellular apoptosis through the p53–PUMA pathway in GBM cell lines. Western blot analysis of apoptosis (PARP, cleavage of caspase 9 and caspase 3), *E2F1*, *p53*, and *PUMA*, a downstream target of p53, in p53 wild-type GBM cells (U87 and DBTRG-05MG) **(A)** and p53 mutant GBM cells (U118MG and LN229) **(B)**. p53 wild-type GBM cells (U87 and DBTRG-05MG) were treated with TMZ (1 mM), VPA (2.5 mM), or TMZ (1 mM) combined with VPA (2.5 mM) for 24 h, and p53 mutant GBM cells (U118MG and LN229) were treated with TMZ (3 mM), VPA (2.5 mM), or TMZ (3 mM) combined with VPA (2.5 mM) for 24 h. **(C, D)** Western blot analysis of apoptosis (PARP, cleavage of caspase 9 and caspase 3), E2F1, p53, and PUMA, a downstream target of p53, in U87 and DBTRG-05MG cells after the knockdown of *p53* with siRNA (Si-p53) followed by TMZ or VPA treatment, as described above. Note that the knockdown of *p53* reduced the apoptosis induced by TMZ combined with VPA in p53 wild-type cell lines.

Since the survival benefit of TMZ combined with VPA treatment was observed in newly diagnosed GBM patients with wild-type *p53* (**Figure 1D**), we hypothesize that VPA may exert its pro-survival effect by enhancing the anticancer activity of TMZ dependent on the *p53* gene status. To determine whether VPA enhances the TMZ-mediated inhibition of GBM cancer cell proliferation and whether *p53* mutation affects susceptibility to GBM, we examined the effect of TMZ alone or in combination with VPA on GBM cancer cell lines with varying p53 status. We found that co-treatment with VPA enhanced the growth inhibitory effect of TMZ in p53 wild-type GBM cells U87 and DBTRG-05MG, but did not significantly affect the growth of p53 mutant GBM cells LN229 and U118MG (**Figures 2A, B** and **Supplementary Figure S7**). This is consistent with the cell cycle analysis showing that combined treatment with TMZ and VPA increased the sub-G1 population in U87 and DBTRG-05MG cells (**Figures 2C, D** **)**, indicating enhanced cellular apoptosis. The effect was not apparent in LN229 and U118MG cells (**Figures 2E, F** **)**. Western blotting analysis confirmed that VPA treatment significantly increased caspase 3 and caspase 9 cleavage in U87 and DBTRG-05MG cells and increased the expression of PUMA, a pro-apoptotic protein downstream of p53 activation, as measured 24 h after TMZ treatment, but not in LN229 and U118MG cells (**Figures 3A, B** and **Supplementary Figure S8**). These results suggest that, in TMZ-induced genotoxic events, VPA increased TMZ cytotoxicity by activating p53 and enhancing the expression of PUMA. To further validate the role of p53 in VPA-induced apoptosis in combination with TMZ, we knocked down *p53* in the U87 and DBTRG-05MG cell lines and evaluated the activation of the apoptosis pathway and the expression of PUMA in response to TMZ or VPA treatment. Knockdown of *p53* expression in both cell lines significantly reduced the activation of the apoptotic pathway, as measured by caspase 9 and caspase 3 activation and enhanced PUMA expression (**Figures 3C, D** **)**. In conclusion, our results show that the effect of VPA on GBM cancer cell proliferation is p53-dependent and that VPA enhances TMZ-induced apoptosis by promoting the expression of *PUMA*, a pro-apoptotic gene downstream of p53.

### Neither *E2F1* nor *HDAC2* Further Enhanced TMZ-VPA-Induced Apoptosis in p53 Wild-Type GBM Cells

**Figure 4:**
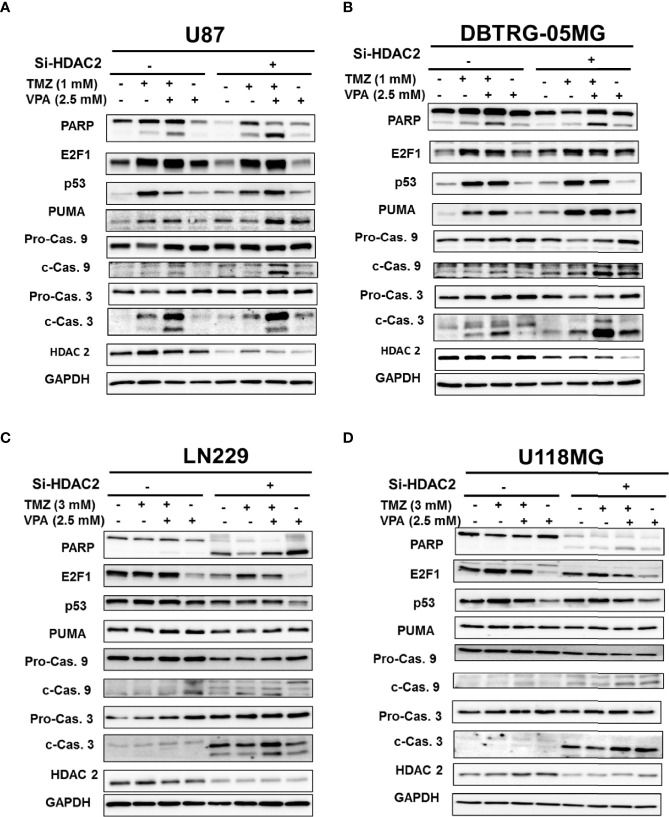
Knockdown of *HDAC2* further enhanced the apoptosis pathway induced by temozolomide (TMZ) combined with valproic acid (VPA) in p53 wild-type cell lines. **(A)** Western blot analysis of apoptosis (PARP, cleavage of caspase 9 and caspase 3), E2F1, p53, and PUMA, a downstream target of p53, in p53 wild-type GBM cells, U87 **(A)** and DBTRG-05MG **(B)**, and in p53 mutant GBM cells, LN229 **(C)** and U118MG **(D)**, after the knockdown of *HDAC2* with siRNA (Si-HDAC2) followed by TMZ or VPA treatment, as described below. p53 wild-type GBM cells (U87 and DBTRG-05MG) were treated with TMZ (1 mM), VPA (2.5 mM), or TMZ (1 mM) combined with VPA (2.5 mM) for 24 h, and p53 mutant GBM cells (U118MG and LN229) were treated with TMZ (3 mM), VPA (2.5 mM), or TMZ (3 mM) combined with VPA (2.5 mM) for 24 h. Note that the knockdown of *HDAC2* induced cellular apoptosis in p53 mutant GBM cells.

We tried to further delineate the mechanisms by which VPA enhanced p53 downstream *PUMA* expression. Previous studies have shown that abundant crosstalk exists between the p53 and E2F1 pathways (38). In genotoxic events, there is extensive crosstalk between the MDM2–p53 and Rb–E2F1 pathways, which cooperate to initiate apoptosis. We found that the expression of *E2F1* was decreased in response to TMZ or TMZ+VPA treatment after *p53* knockdown (**Figures 3C, D** **)**. However, *E2F1* knockdown did not affect the activation of the apoptosis pathway or the expression of PUMA induced by VPA+TMZ treatment (**Supplementary Figure S9**). The results suggest that *E2F1* activation was modulated by p53 activation, but was not necessary for TMZ+VPA to exert its pro-apoptotic function. Additionally, several studies indicated that the degradation of HDAC2 is one of the anticancer actions of VPA (28, 39) and that knockdown of *HDAC2* enhances the sensitivity of GBM cancer cells to TMZ (40). Therefore, we examined the expression and the effect of HDAC2 on the apoptosis-enhancing ability of VPA+TMZ treatment. We found that HDAC2 expression did not decrease after VPA treatment for 24 h alone or in combination with TMZ (**Figures 4A, B** **)**. Additionally, knockdown of *HDAC2* did not abrogate but further enhanced the pro-apoptotic effect of VPA+TMZ co-treatment in either p53 wild-type GBM cell line, as inferred from the increased PARP cleavage and caspase 9 and caspase 3 activation (**Figures 4A, B** **)**. A synergistic effect was not observed in either p53 mutant GBM cell line (**Figures 4C, D** **)**. Interestingly, knockdown of *HDAC2* in LN229 and U118MG p53 mutant cells induced cellular apoptosis without TMZ or VPA treatment (**Figures 4C, D** **)**. Our results indicate that VPA may not exert its effect by inhibiting E2F1 activation or HDAC2 activity/expression. HDAC2 inhibition may induce apoptosis in p53 mutant cells *via* other signaling pathways.

## Discussion

Glioblastoma (GBM) has been shown to harbor great genetic, epigenetic, and gene expression heterogeneities in both interpersonal and intratumor tissues (4–6, 41). GBM arises *de novo* (primary GBM) or *via* the dedifferentiation of lower-grade glioma (secondary GBM) (42). While distinct mutations are predominant in each subtype, alterations of the tumor suppressor *p53* are the most common (43). These molecular alterations of GBM significantly affect tumor susceptibility to chemotherapy and, thus, patient prognosis (19–21). It has been shown that the expressions of p53 in newly diagnosed and recurrent GBM patients are inconsistent and can be altered upon recurrence (44, 45). Whether *p53* mutation affects GBM sensitivity to chemotherapy and prognosis remains controversial (46–49). In this study, our results indicate that VPA enhances TMZ cytotoxicity by promoting apoptosis through enhancing p53 pathway activation and increasing the expression of its downstream target gene, *PUMA*. Wild-type p53 expression in GBM cells is necessary for VPA to exert its function. Screening of *p53* mutations may help identify GBM patients who will benefit from a combined VPA and TMZ treatment.

The life expectancy of GBM patients is 12–18 months, despite advances in diagnosis and treatment (50–52). Several known prognostic factors include age, preoperative functional status, history of seizure, tumor location and size, extent of surgery, use of radiotherapy, and *IDH* mutation (52–56). In our study, the median survival of newly diagnosed GBM patients was 15.67 ± 1.42 months. Younger age (<65 years), seizure history, and long-term VPA treatment (>30 days) were associated with favorable outcomes (**Supplementary Figures S1A, C** and **Figure 1A**). The results are roughly concordant with other studies (52, 54). On the other hand, the median survival of recurrent GBM patients was 13.10 ± 2.34 months, which was not significantly different from that of newly diagnosed patients (*p* = 0.300). Since all our recurrent GBM patients underwent surgical excision, it is likely that the included patients were in better physical condition capable of undergoing surgery and had tumors that were presumably located at favorable locations suited for surgical intervention, thus improving the results. When combining newly diagnosed and recurrent GBM patients, only VPA treatment was statistically significantly associated with improved survival (**Figure 1A**).

VPA is used to treat seizures in our hospital. Although it has been shown that long-term use of anticonvulsants in seizure-free patients adds no clinical benefits (57, 58), some physicians in our institute were used to keeping anticonvulsants for patients who had symptoms suspicious of complex partial seizures or absence of seizures until the diagnosis was excluded. Therefore, in our dataset, there were some patients who had no definite seizure diagnosis, but who received VPA for more than 1 month (**Table 2**). Although long-term VPA treatment was associated with improved survival in the single variate analysis (**Figure 1A**), multivariate Cox regression analysis did not indicate VPA use as an independent prognostic factor (**Supplementary Tables S1** and **S2**). Since VPA is used to treat seizures, which in itself is a favorable prognostic factor, evaluation of the VPA effect on the survival of GBM patient is confounded by the presence of seizures. Furthermore, there appeared to be more female patients in our long-term VPA group (**Table 2**). Gender alone does not affect treatments given to patients in our hospital. Although our seizure treatment did not differentiate between males or females, it was not shown in our study that gender significantly affected the results of GBM survival or VPA treatment (**Supplementary Figure S1B**), which indicates that there may be some degree of selection bias in our dataset. Further analysis indicated that VPA treatment appeared to be effective in newly diagnosed GBM patients and in younger patients (**Figures 1B, C** **)**, but not in recurrent GBM or in patients over 65 years old (**Supplementary Figures S1D, E**). This result is also concordant with previous studies (59, 60). Interestingly, in our analysis, the benefit of long-term VPA treatment did not reach statistical significance in either the seizure or no-seizure group but in the combined group (**Supplementary Table S3**), indicating that the presence or absence of seizures does not significantly impact the effect of VPA. Kuo et al. reported that VPA does not improve survival in patients who are seizure-free (59). This discrepancy is likely caused by our small sample size and the patient variability and requires further investigation.

The *MGMT* promoter methylation status has long been recognized as a prognostic factor of GBM patient survival (61, 62). However, it was not routinely examined in our hospital because the test was not covered by our National Health Insurance program. The Clinical Pathology Department in our institute used IHC staining to detect MGMT expression, which is known to have low specificity and undetermined thresholds and may not have a strong correlation with the outcomes of GBM patients (63–65). In this study, we did not have enough resources or patient samples to perform methylation-specific PCR. Our analysis did not demonstrate a survival benefit in either MGMT staining-positive or MGMT staining-negative patients (**Supplementary Figure S2**). We also did not detect a pro-survival effect of VPA treatment in either MGMT staining-positive or MGMT staining-negative patients (**Supplementary Figures S3A, D**). In newly diagnosed MGMT-negative GBM patients, there appeared to be a trend toward improved survival in the long-term VPA treatment group, but this trend did not reach statistical significance (*p* = 0.052) (**Supplementary Figure S3E**). Interestingly, there are some reports indicating that VPA may enhance TMZ sensitivity by downregulating MGMT expression (30). Roos et al. reported that GBM cells with functionally intact *p53* genes were more sensitive to TMZ treatment due to the activation of the Fas/CD95/APO-1 receptor and the subsequent apoptosis triggered by *O* ^6^-methylguanine, a product of TMZ-induced DNA damage (66). Further studies evaluating the effect of *MGMT* promoter methylation on VPA treatment and its interaction with the p53 pathway may help better identify VPA-responsive patients. In conclusion, our data indicate a possible survival benefit of prolonged VPA treatment in younger, newly diagnosed GBM patients.

The molecular alterations of GBM have been shown to significantly affect tumor susceptibility to chemotherapy and, thus, patient prognosis (19–21). In this study, *p53* mutation was detected in 11 out of the 85 patients sampled. The mutational status of *p53* did not significantly affect patient survival (**Supplementary Figure S5**), which is concordant with previous studies (67). We also found that TMZ combined with long-term VPA treatment was effective in GBM patients with wild-type *p53* (**Figure 1D**). Additionally, genetic alterations affecting the function of the p53 pathway, such as CDKN2A/ARF deletion or MDM2/MDM4 amplification, could be present in ~85% of all GBM patients (16) and had similar effects on the pro-survival effect of VPA treatment. Whether these genetic alterations that impair the p53 pathway activation affect VPA-induced TMZ potentiation requires further study. Since only 11 *p53* mutant samples were identified (**Table 3**), the study may be limited by its small sample size, patient heterogeneity, and possible selection bias. However, we were able to demonstrate that VPA enhances TMZ cytotoxicity by enhancing apoptosis *via* the p53 pathway and the expression of the downstream target, *PUMA* (**Figures 3A, B** and **Supplementary Figure S8**) using p53 wild-type and p53 mutant human GBM cells.

p53 is commonly activated in response to DNA damage, genotoxicity, oncogene activation, aberrant growth signals, and hypoxia, all of which are events that can be encountered during carcinogenesis (48). p53 upregulated modulator of apoptosis (PUMA), a Bcl-2 homology 3 (BH3)-only pro-apoptotic Bcl-2 family member, was identified as a molecule that directly mediates p53-associated apoptosis (68). The PUMA protein associates with the mitochondria and induces apoptosis much earlier than the apoptosis that results from the exogenous expression of p53 when it is overexpressed in various cell lines (69). Previous studies have shown that PUMA overexpression results in massive apoptosis in GBM cells with wild-type or mutant p53, indicating that it is a therapeutic tool for GBM (70). Additionally, PUMA has been shown to increase the drug sensitivity of TMZ-resistant cells; thus, PUMA may be a suitable target for intervention to improve the therapeutic efficacy of TMZ (71). Here, we found that PUMA was further induced in p53 wild-type GBM cells, U87 cells, and DBTRG-05MG cells (**Figure 3A**). In our study, TMZ treatment induced p53 activation and apoptosis in p53 wild-type GBM cells, which was further enhanced by VPA treatment. For the *in vitro* experiments with GBM cells, we used TMZ at concentrations of 1 and 3 mM, based roughly on the IC_50_ at 24 h (**Figure 2**). This TMZ concentration induces cell death mainly *via* non-repaired *N*-alkylations, while for *O* ^6^-methylguanine-induced apoptosis, much lower doses are sufficient, which are in the range of 1–50 μM (72). Under clinical conditions, the tissue concentration of TMZ is approximately 1 μg/ml (5.2 μM) and the serum concentration about 15 μg/ml (78 μM) (73, 74). In this concentration range, the main treatment effect is achieved *via O* ^6^-methylguanine-induced DNA double-strand breaks and subsequent apoptosis. Whether the findings reported here are clinically relevant requires further elucidation. Additionally, there were some inconsistent reports of *p53* gene activity in LN229 cells (75, 76). The majority agrees that LN229 retains at least partial p53 activity despite the mutation. In our study, we did find a missense mutation at exon 4. Treatment with TMZ failed to induce PUMA expression in LN229 cells (**Figure 3B**), suggesting at least a possible partial loss of p53 function. We also demonstrated that the knockdown of *p53* abrogated the expression of PUMA and the cleavage of caspase 9, caspase 3, and PARP in p53 wild-type GBM cells treated with TMZ+VPA (**Figures 3C, D** **)** and reversed the pro-apoptotic effect of VPA in TMZ treatment, indicating that VPA enhanced TMZ-induced cell apoptosis *via* p53–PUMA pathway activation.

Several mechanisms for the anticancer effect of VPA have been proposed, including inhibition of HDAC, alteration of the chromatin structure, disruption of DNA repair pathways or redox regulation, and induction of autophagy (11, 27–30). Xie et al. reported that VPA attenuates the immunosuppressive function of myeloid-derived suppressor cells and may potentially improve the antitumor activity of CD8^+^ T cells (77). In malignant melanoma cells, VPA was shown to enhance IFN-β-induced caspase 8 expression, thus improving its response to TMZ treatment (78). In murine limb organogenesis, VPA was shown to induce p53 hyperacetylation through its HDAC inhibitor activity, thus enhancing p53 target gene expression (79). Among these mechanisms, VPA, as an HDAC inhibitor, has been widely recognized. It induces HDAC2 degradation and inhibits HDAC2 activity (28, 39). However, our results did not show a reduction in HDAC2 expression subsequent to VPA treatment (**Figure 4**). Knockdown of HDAC2 further enhanced TMZ+VPA-induced cellular apoptosis in p53 wild-type GBM cells (**Figures 4A, B** **)**. Interestingly, HDAC2 silencing induced cellular apoptosis in p53 mutant GBM cells (**Figures 4C, D** **)**, which is consistent with previous studies showing that silencing *HDAC2* can suppress the proliferation of GBM cells (40). On the other hand, several potent HDAC inhibitors that entered clinical trials in recent years, such as vorinostat (SAHA) or trichostatin A (TSA), failed to demonstrate a significant survival benefit to patients in phase II clinical trials, either as single agents or in combination with standard TMZ treatment (80–82). This is consistent with our results that only VPA, but not SAHA or TSA, enhanced TMZ-induced apoptosis in U87 cells (**Supplementary Figure S10**). These results together suggest that VPA may not exert its effect by inhibiting HDAC2 activity or expression, and HDAC2 inhibition may potentiate the effect *via* other signaling pathways.

## Conclusion

Our results indicate that the survival benefit of the combination regimen of TMZ and VPA in GBM patients is dependent on their *p53* mutation status. In cellular models, our results show that VPA enhanced the antineoplastic effect of TMZ by enhancing apoptosis *via* activation of the p53 pathway and increasing the expression of its downstream pro-apoptotic protein, PUMA. Taken together, wild-type *p53* may serve as an indicator of the effectiveness of a combined TMZ+VPA treatment in GBM.

## Data Availability Statement

The original contributions presented in the study are included in the article/**Supplementary Material**. Further inquiries can be directed to the corresponding author.

## Ethics Statement

The studies involving human participants were reviewed and approved by Institutional Review Board of Chang-Gung Medical Foundation (IRB#: 201701979B0). Written informed consent for participation was not required for this study in accordance with the national legislation and the institutional requirements.

## Author Contributions

H-CT, K-CW, and H-TW designed, performed research, and analyzed data. H-CT, K-CW, P-YC, C-YH, K-TC, Y-JL, and Y-RC recruited, collected clinical specimens, and analyzed clinical data. H-CT and H-WC performed the experiments. H-CT and H-TW wrote the paper. All authors contributed to the article and approved the submitted version.

## Funding

This work was funded by grants from the Ministry of Science and Technology, Taiwan [NHRI-EX110-11027PI, 109-2320-B-010-024 (H-TW) and 107-2320-B-182-044 (H-CT)], and Chang-Gung Memorial Hospital [CMRPG3K1441 (H-CT)].

## Conflict of Interest

The authors declare that the research was conducted in the absence of any commercial or financial relationships that could be construed as a potential conflict of interest.

## Publisher’s Note

All claims expressed in this article are solely those of the authors and do not necessarily represent those of their affiliated organizations, or those of the publisher, the editors and the reviewers. Any product that may be evaluated in this article, or claim that may be made by its manufacturer, is not guaranteed or endorsed by the publisher.
