# Supplementary material for: Valproic Acid Enhanced Temozolomide-Induced Anticancer Activity in Human Glioma Through the p53–PUMA Apoptosis Pathway
Source: Front Oncol. 2021 Oct 1;11:722754. doi: 10.3389/fonc.2021.722754 (PMC8518553; doi:10.3389/fonc.2021.722754)
Supplement: Supplementary file 1 [file DataSheet_1.docx]

**Supplementary Figures**

**
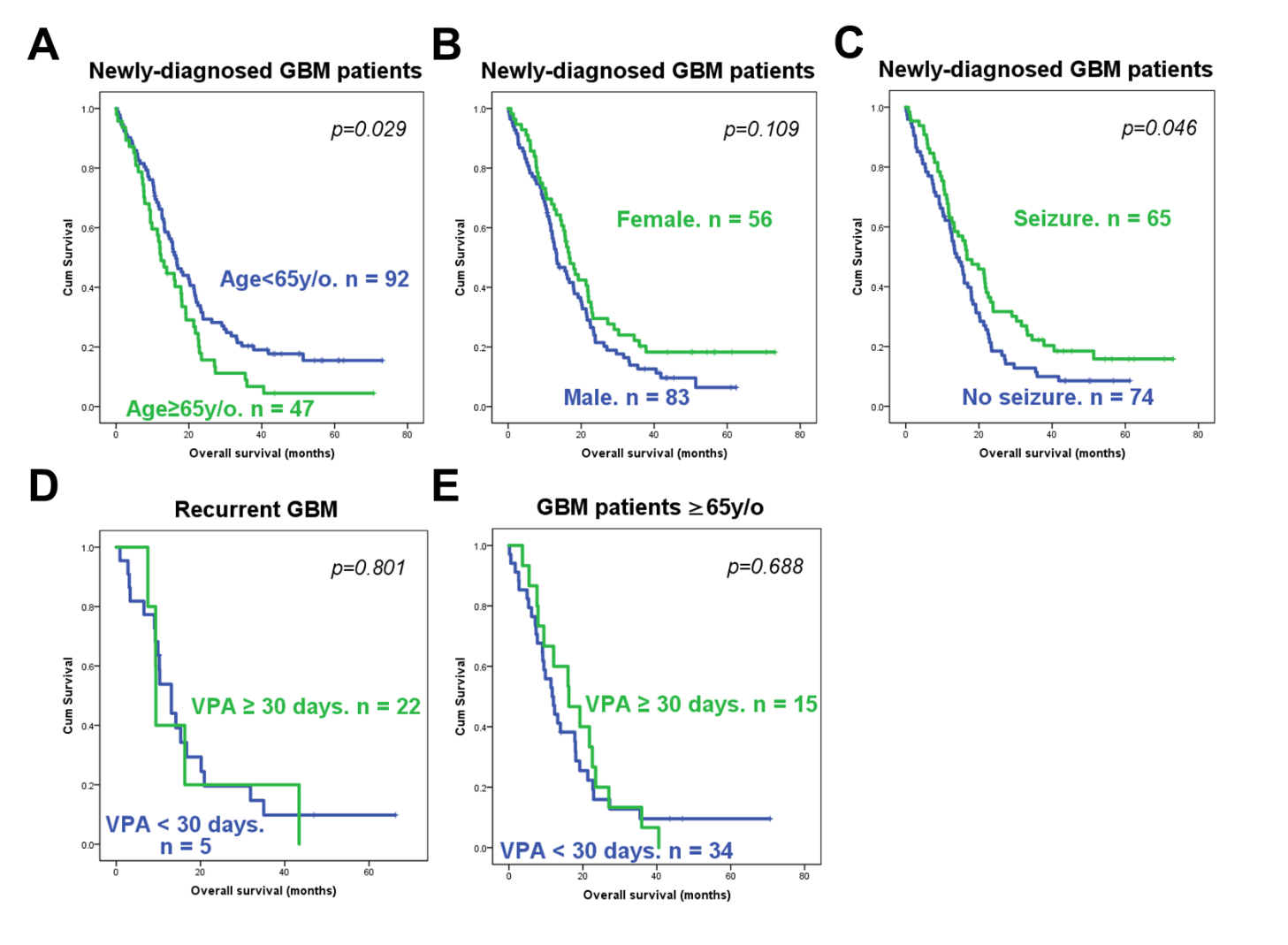
**

**Supplementary Figure 1.** **Kaplan-Meier analysis of GBM patients.** Survival plot of newly-diagnosed GBM patients (n=139) according to (A) age, (B) gender and (C) seizure group. (D) Survival plot of all recurrent GBM patients (n=27) and (E) older GBM patients (≥65 y/o) (n=49) according to VPA treatment. P-value was calculated using log-rank test in SPSS statistical analysis software.

**
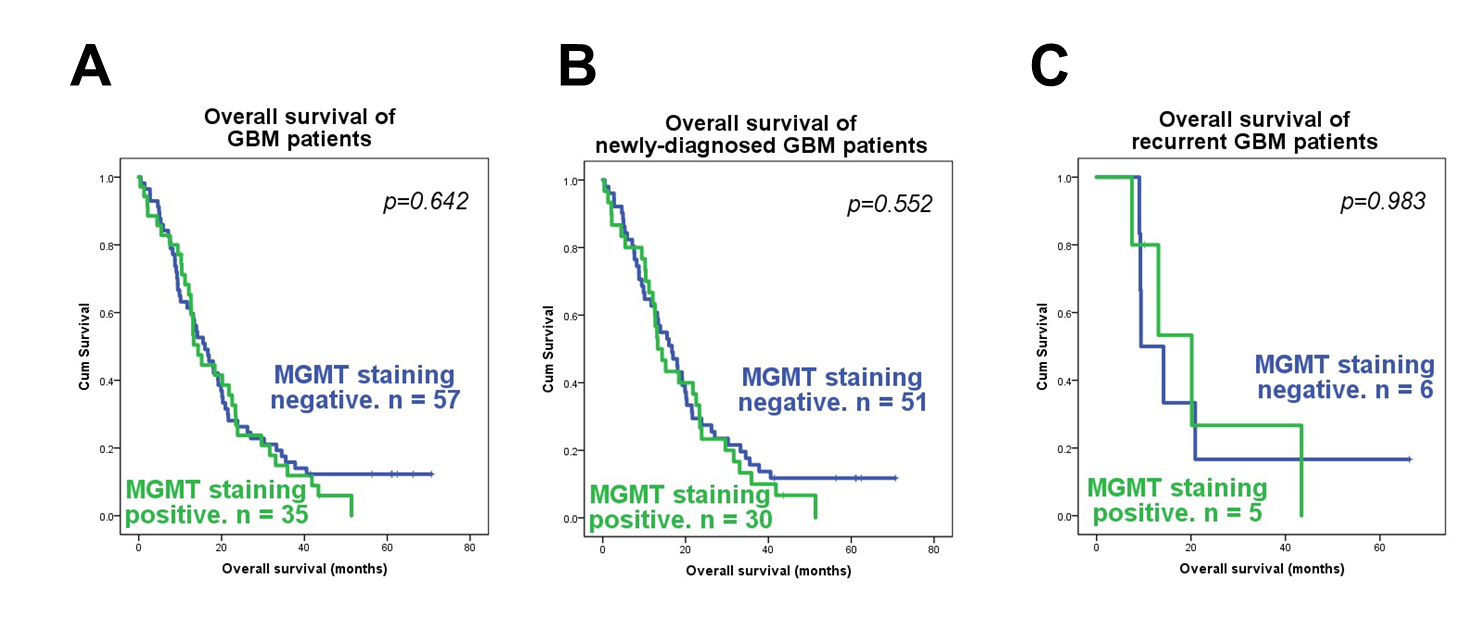
**

**Supplementary Figure 2. Kaplan-Meier analysis of GBM patients according to IHC staining of O6-methylguanine DNA methyltransferase (MGMT).** Survival plot of (A) all GBM patients (n=92), (B) newly-diagnosed GBM patients (n=81) or (C) recurrent GBM patients (n=11) according to MGMT staining. P-value was calculated using log-rank test in SPSS statistical analysis software.

**
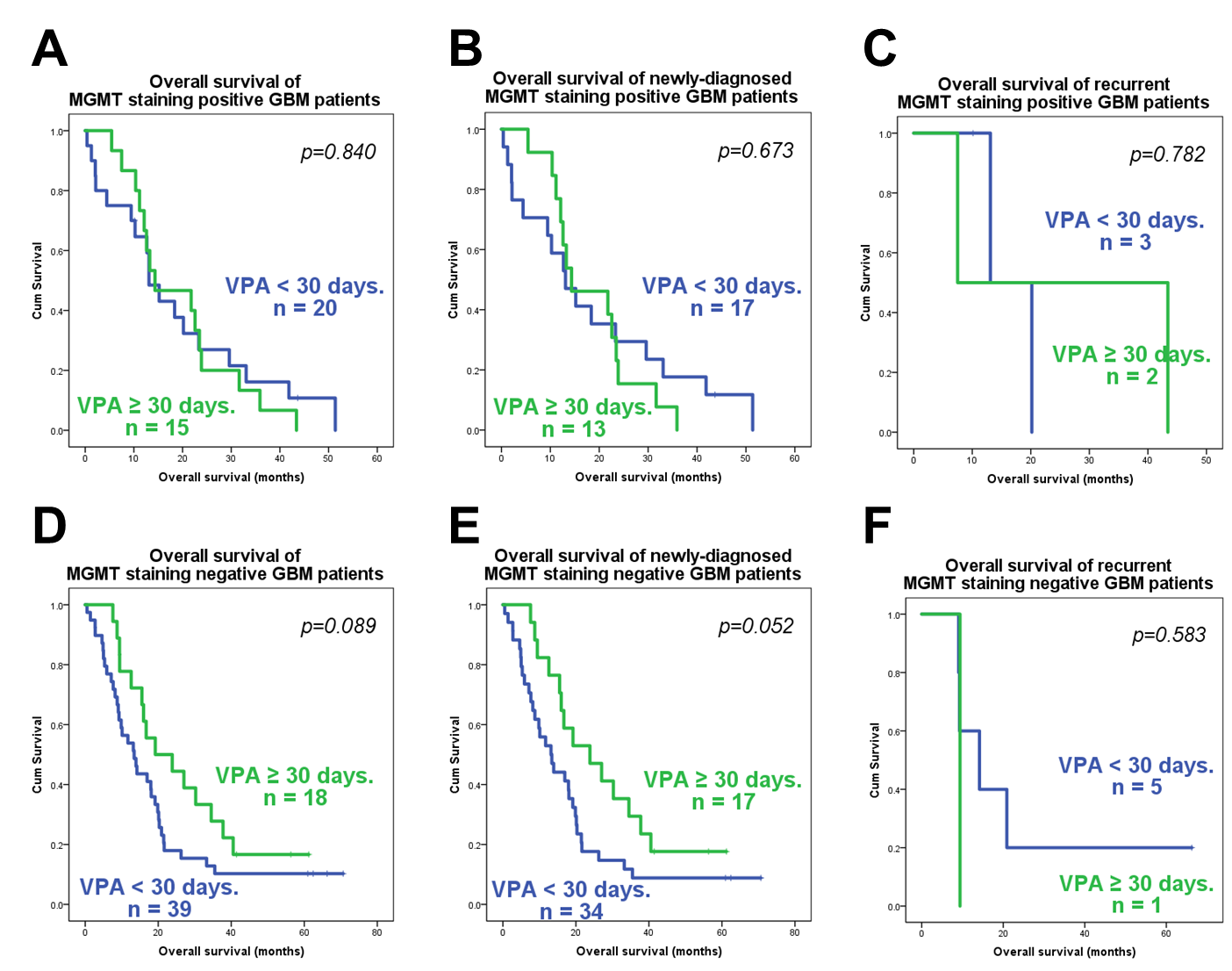
**

**Supplementary Figure 3. Kaplan-Meier analysis of GBM patients according to VPA treatment.** Survival plot of (A) all MGMT-staining positive GBM patients (n=35), (B) MGMT-staining positive newly-diagnosed GBM patients (n=30), (C) MGMT-staining positive recurrent GBM patients (n=5), (D) all MGMT-staining negative GBM patients (n=57), (E) MGMT-staining negative newly-diagnosed GBM patients (n=51) or (F) MGMT-staining negative recurrent GBM patients (n=6). P-value was calculated using log-rank test in SPSS statistical analysis software.

**
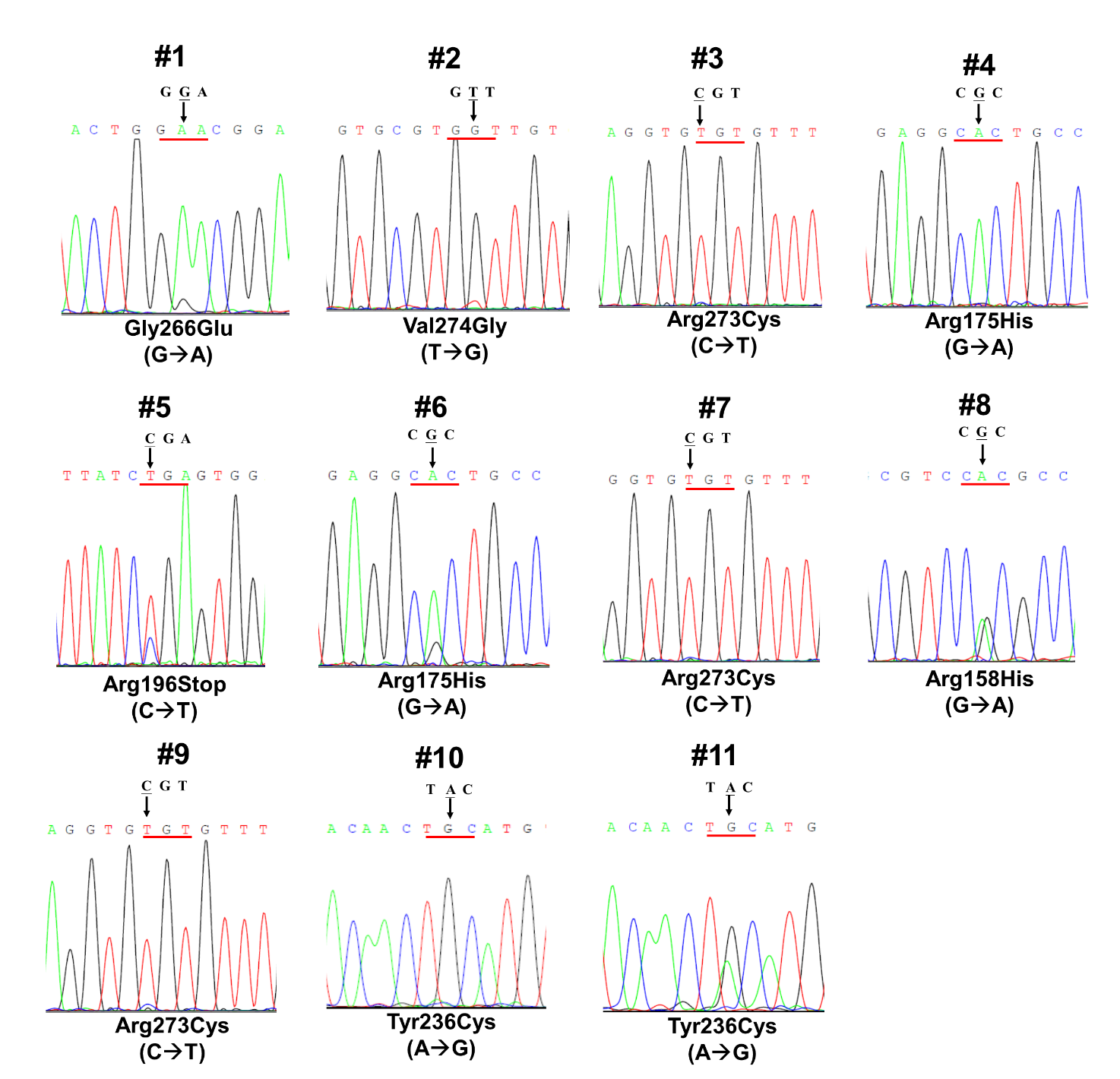
**

**Supplementary Figure 4. Representative diagram of DNA sequencing of exon 5-9 of *p53* mutation in 11 glioma patients.** Genomic DNA was extracted from frozen tissues of glioma patients and exon 5-9 of the p53 was PCR-amplified from tumor DNA and mutations were analyzed DNA sequencing as described in Materials and methods.


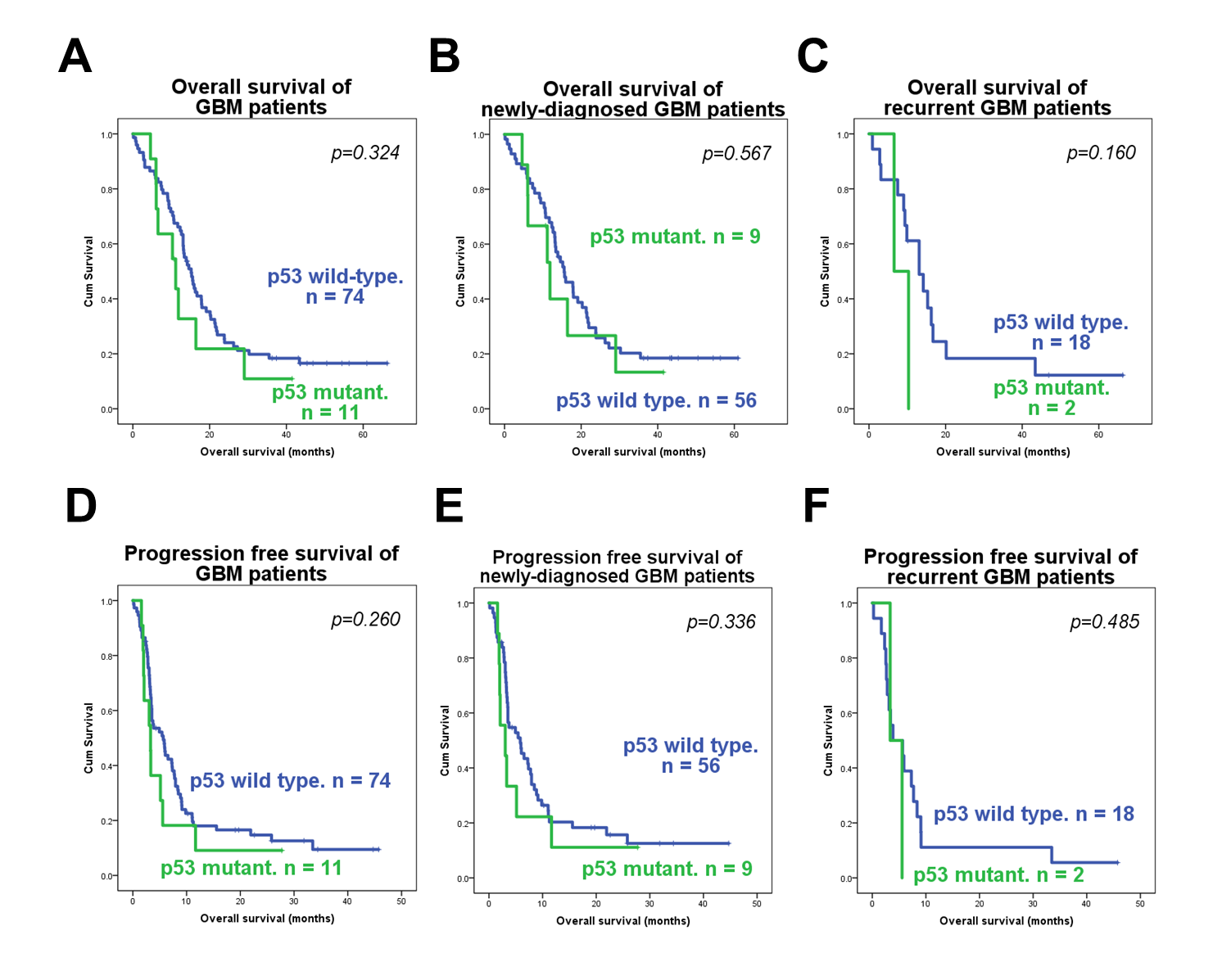


**Supplementary Figure 5. Kaplan-Meier analysis of GBM patients with p53 sequencing results.** Overall survival plot of (A) all GBM patients (n=85), (B) newly-diagnosed GBM patients (n=65) or (C) recurrent GBM patients (n=20) according to p53 status. Progression free survival plot of (D) all GBM patients (n=85), (E) newly-diagnosed GBM patients (n=65) or (F) recurrent GBM patients (n=20) according to p53 status. P-value was calculated using log-rank test in SPSS statistical analysis software.

**
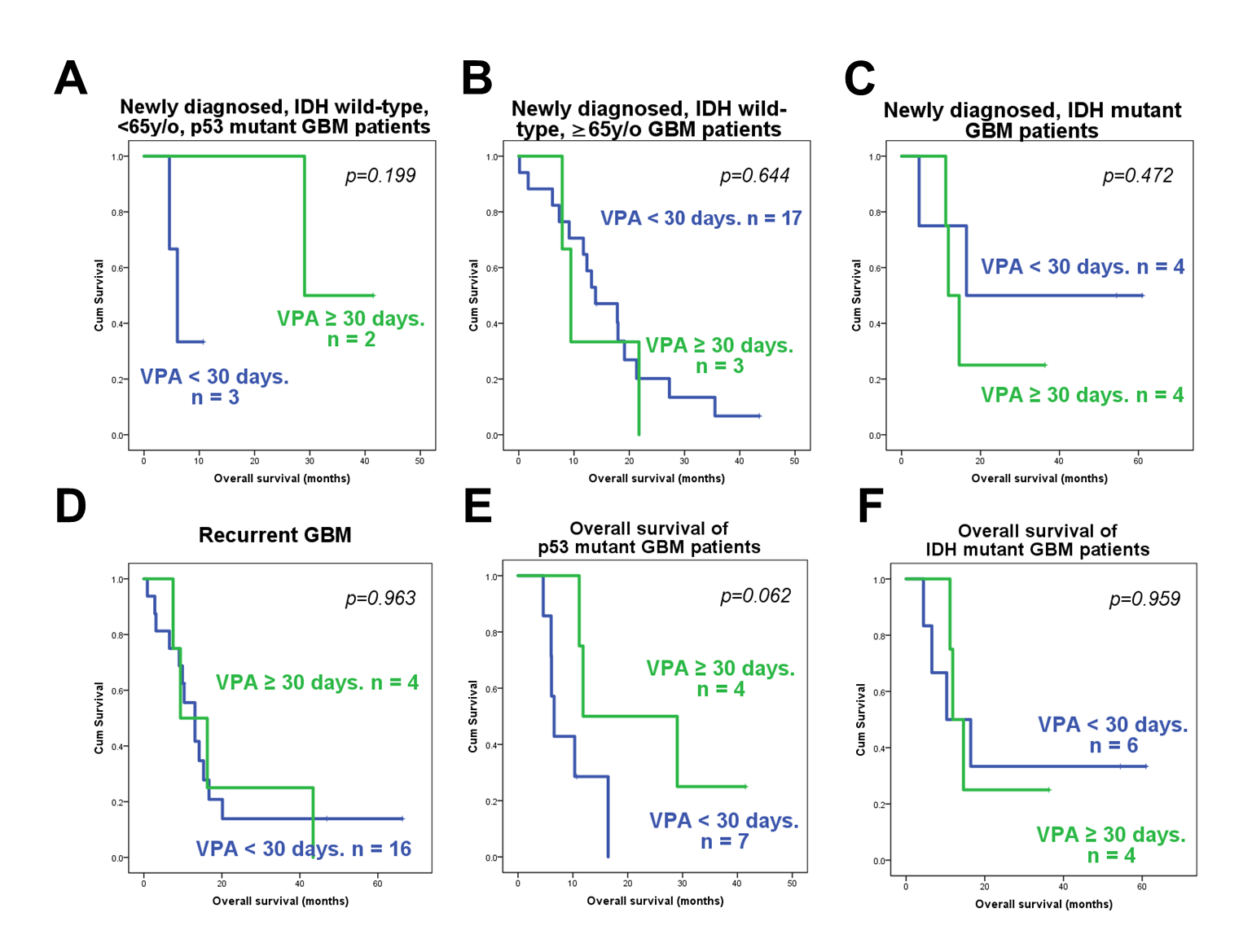
**

**Supplementary Figure 6. Kaplan-Meier survival analysis of survival of newly diagnosed and recurrent GBM patients with different mutation status.** Survival plot of (A) younger newly-diagnosed GBM patients with wild type IDH and mutant p53 genes, (B) older newly-diagnosed GBM patients with wild type IDH gene, (C) newly-diagnosed GBM patients with mutant IDH gene, (D) recurrent GBM, (E) p53 mutant GBM, (F) IDH1 mutant GBM. P-value was calculated using log-rank test in SPSS statistical analysis software.

**
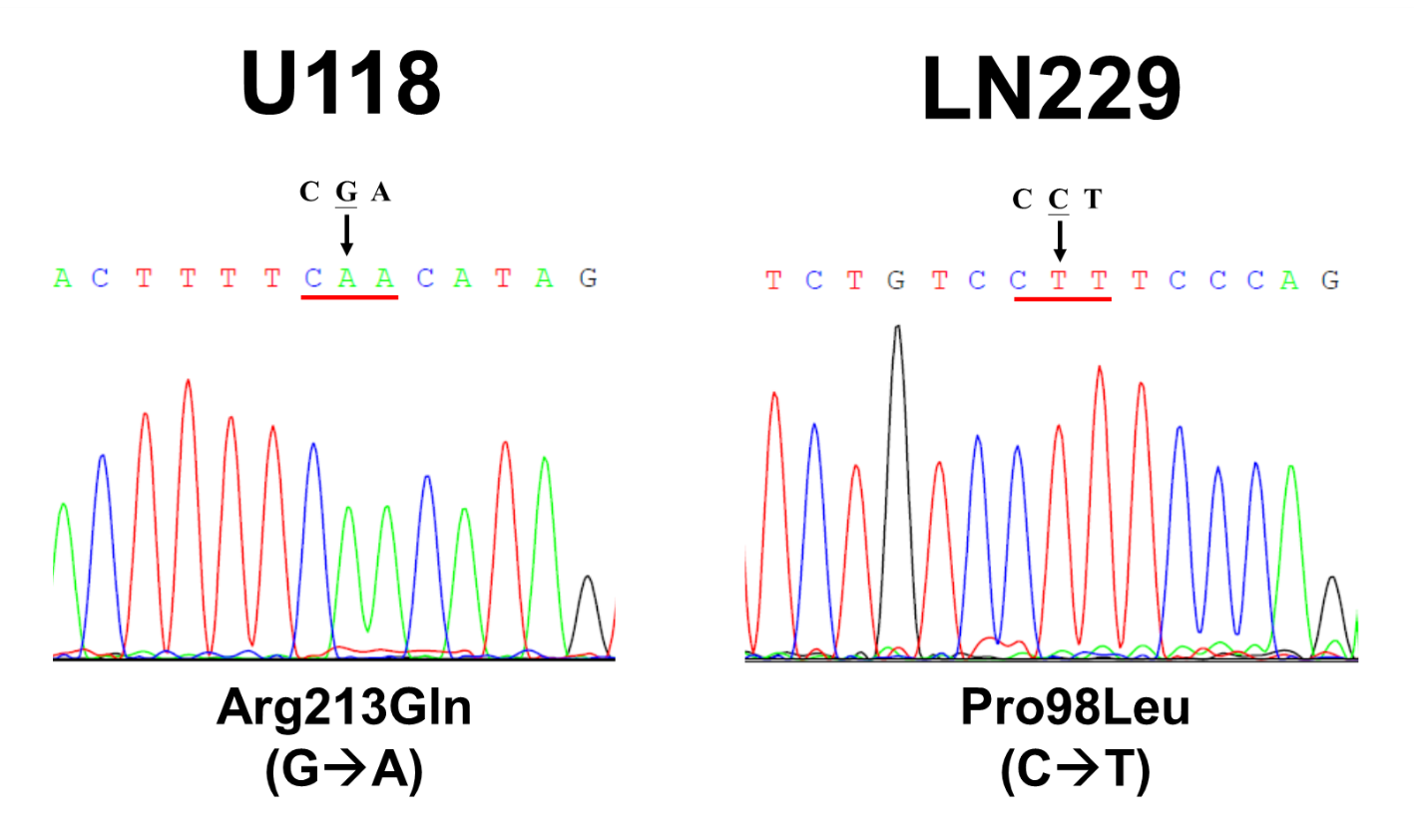
**

**Supplementary Figure 7. Representative diagram of DNA sequencing of *p53* mutation in human glioma cell lines, U118 and LN229.** Genomic DNA was extracted from glioma cell lines and exon 4-9 of the p53 was PCR-amplified from tumor DNA and mutations were analyzed DNA sequencing as described in Materials and methods.

**
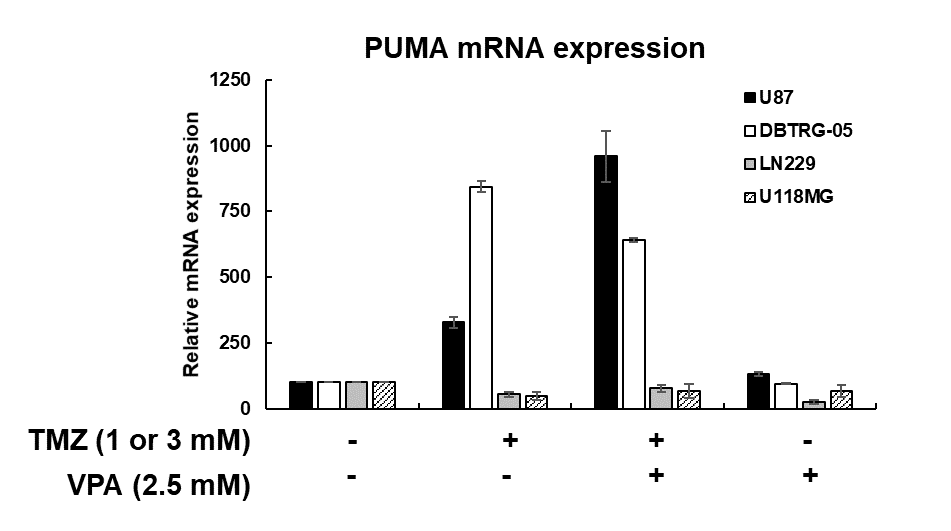
**

**Supplementary Figure 8. Relative mRNA expression of PUMA in GBM cells treated with temozolomide (TMZ) in combined with valproic acid (VPA).** mRNA expression of PUMA in p53 wild type GBM cells (U87 and DBTRG-05MG) and p53 mutant GBM cells (U118MG and LM229) was analyzed using real time RT-PCR analysis as describe in Materials and methods. p53 wild-type GBM cells (U87 and DBTRG-05MG) were treated with TMZ (1 mM), VPA (2.5 mM) or TMZ (1 mM) combined with VPA (2.5 mM) for 24 h, and p53 mutant GBM cells (U118MG and LN229) were treated with TMZ (3 mM), VPA (2.5 mM) or TMZ (3 mM) combined with VPA (2.5 mM) for 24 h.


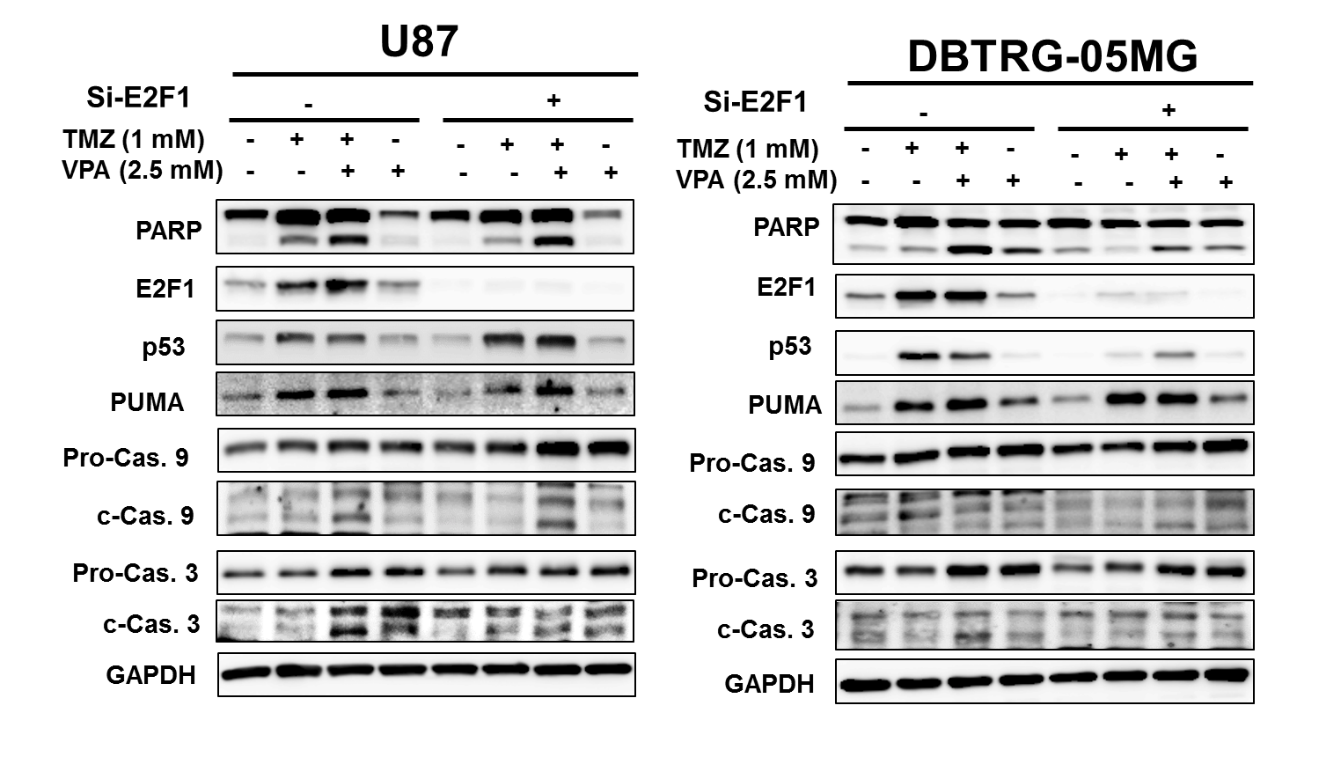


**Supplementary Figure 9. Knockdown of E2F1 did not affect apoptosis pathway induced by TMZ combined with VPA in p53 wild type cell lines.** Western blot analysis of apoptosis (PARP, cleavage of caspase 9 and caspase 3), E2F1, p53, PUMA, a downstream target of p53, in p53 wild type GBM cells, U87 and DBTRG-05MG after knockdown of E2F1 with siRNA.


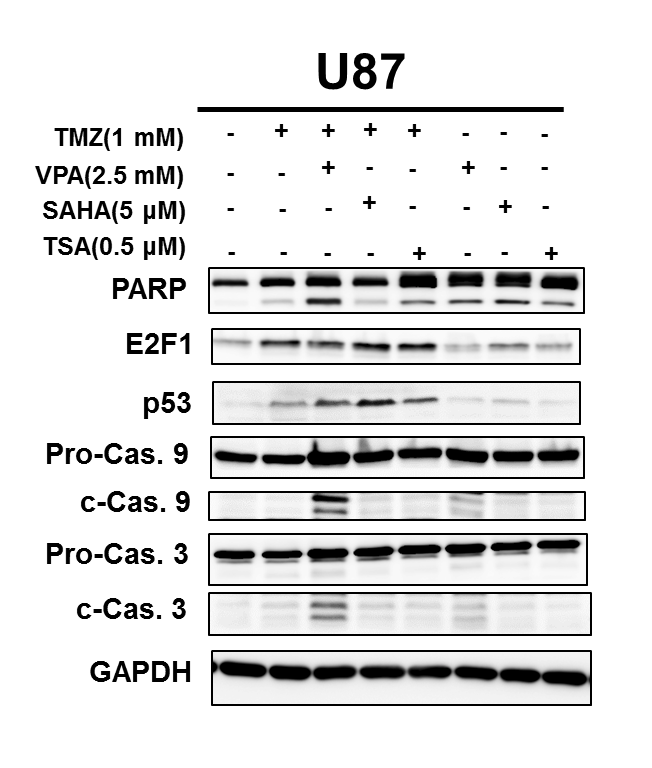


**Supplementary figure 10.** **VPA, but not HDAC inhibitors enhanced TMZ induced apoptosis in U87 cells.** Western blot analysis of apoptosis (PARP, cleavage of caspase 9 and caspase 3), E2F1, p53 in U87 after treatment of TMZ in combined with VPA (2.5 mM) or HDAC inhibitors, SAHA (5 μM) or TSA (0.5 μM) for 24 h.

**Supplementary Tables**

**Supplementary Table 1. Cox regression analysis for clinical characteristics of all GBM patients.**

| Overall survival of all GBM patients | | | |
| --- | --- | --- | --- |
| Variable | HR | 95% CI | P value |
| Age group (<65 y/o vs ≥ 65 y/o) | 0.802 | 0.558-1.154 | 0.235 |
| Sex (Male vs Female) | 1.229 | 0.871-1.735 | 0.241 |
| Seizure (No seizure vs Seizure) | 1.150 | 0.810-1.634 | 0.434 |
| VPA treatment (<30 days vs ≥ 30 days) | 1.440 | 0.996-2.081 | 0.053 |

**Supplementary Table 2. Cox regression analysis for clinical characteristics of newly-diagnosed GBM patients.**

| Overall survival of newly-diagnosed GBM patients | | | |
| --- | --- | --- | --- |
| Variable | HR | 95% CI | P value |
| Age group (<65 y/o vs ≥ 65 y/o) | 0.728 | 0.494-1.075 | 0.111 |
| Sex (Male vs Female) | 1.280 | 0.874-1.875 | 0.205 |
| Seizure (No seizure vs Seizure) | 1.255 | 0.848-1.858 | 0.255 |
| VPA treatment (<30 days vs ≥ 30 days) | 1.458 | 0.978-2.174 | 0.064 |

**Supplementary Table 3. Overall survival of different GBM patients with long-term and short-term valproic acid treatment.**

|  |  | VPA treatment | | | P value |
| --- | --- | --- | --- | --- | --- |
|  |  | Short-term  (no or < 30 days) | | Long-term  (≥ 30 days) |  |
|  | Count | 110 | | 56 |  |
|  |  | Months  (Mean ± SD) | Months  (Mean ± SD) | |  |
| Median survival (months) | | 13.10±1.05 | 21.37±3.49 | | 0.012* |
| Age (years) | < 65 y/o | 13.27±1.72 | 22.00±4.01 | | 0.009* |
|  | ≥65 | 11.70±1.82 | 16.20±4.57 | | 0.688 |
| Gender | Male | 13.10±0.83 | 16.70±4.30 | | 0.157 |
|  | Female | 15.20±3.39 | 21.90±4.29 | | 0.092 |
| Seizure status | Yes | 11.70±1.69 | 22.00±4.18 | | 0.136 |
|  | No | 13.10±1.58 | 16.27±2.80 | | 0.087 |
| Newly/ Recurrent | Newly diagnosed | 13.10±1.64 | 21.9±3.33 | | 0.008* |
|  | Recurrent | 13.10±2.11 | 9.40±0.04 | | 0.801 |
| *P<0.05, the chi-square statistic is significant at the 0.05 level. | | | | | |

**Supplementary Table 4. TP53 mutations detected in individual glioma samples.**

| Patient No. | Amino acid change | Mutation | Type of mutation |
| --- | --- | --- | --- |
| #1 | G266E | GGA🡪GAA | missense |
| #2 | V274G | GTT🡪GGT | missense |
| #3 | R273C | CGT🡪TGT | missense |
| #4 | R175H | CGC🡪CAC | missense |
| #5 | R196Stop | CGA🡪TGA | nosense |
| #6 | R175H | CGC🡪CAC | missense |
| #7 | R273C | CGT🡪TGT | missense |
| #8 | R158H | CGC🡪CAC | missense |
| #9 | R273C | CGT🡪TGT | missense |
| #10 | Y236C | TAC🡪TGC | missense |
| #11 | Y236C | TAC🡪TGC | missense |
